# BK Virus and Cytomegalovirus Coinfections in Kidney Transplantation and Their Impact on Allograft Loss

**DOI:** 10.3390/jcm10173779

**Published:** 2021-08-24

**Authors:** Sabina Herrera, Javier Bernal-Maurandi, Frederic Cofan, Pedro Ventura, Maria Angeles Marcos, Laura Linares, Genoveva Cuesta, Fritz Diekmann, Asunción Moreno, Marta Bodro

**Affiliations:** 1 Infectious Diseases Department, Institut d’Investigacions Biomèdiques August Pi i Sunyer (IDIBAPS), University of Barcelona and Hospital Clinic, 08036 Barcelona, Spain; sherrera@clinic.cat (S.H.); yavierb@hotmail.com (J.B.-M.); lalinares@clinic.cat (L.L.); amoreno@clinic.cat (A.M.); 2 Facultad de Medicina, Universidad de Alcalá, Alcalá de Henares, 28805 Madrid, Spain; 3 Department of Nephrology and Renal Transplantation, Institut d’Investigacions Biomèdiques August Pi i Sunyer (IDIBAPS), University of Barcelona and Hospital Clinic, 08036 Barcelona, Spain; fcofan@clinic.cat (F.C.); pventura@clinic.cat (P.V.); fdiekman@clinic.cat (F.D.); 4 Microbiology Department, Institut d’Investigacions Biomèdiques August Pi i Sunyer (IDIBAPS), University of Barcelona and Hospital Clinic, 08036 Barcelona, Spain; mmarcos@clinic.cat (M.A.M.); gcuesta@clinic.cat (G.C.)

**Keywords:** BK virus, cytomegalovirus, kidney transplantation, allograft loss

## Abstract

We aimed to ascertain the interaction and effects of combined reactivations of BK virus and cytomegalovirus on kidney graft function. All consecutive kidney transplant recipients (KTR) between 2003 and 2016 were included. Of 1976 patients who received a kidney transplant, 23 (1.2%) presented BKV-associated nephropathy (BKVAN). Factors independently associated with BKVAN were diabetes mellitus (odds ratios (OR) 3.895%, confidence intervals (CI) (1.4–10.5)), acute allograft rejection (OR 2.8 95%, CI (1.1–7.6)) and nephrostomy requirement (OR 4.195%, CI (1.3–13)). Cytomegalovirus infection was diagnosed in 19% of KTR patients. Recipients with BKVAN presented more frequently with cytomegalovirus (CMV) infection compared to patients without BKVAN (39% vs. 19%, *p* = 0.02). Acute allograft rejection (OR 2.95%, CI (1.4–2.4)) and nephrostomy requirement (OR 2.95%, CI (1.2–3)) were independently associated with CMV infection. Sixteen patients (69%) with BKVAN had graft dysfunction at one-year post-transplant and eight of them (35%) lost their graft. Patients presenting with BKVAN and graft loss presented more frequently a cytomegalovirus infection (OR 2.295%, CI (1.3–4.3)). In conclusion, we found a relation between CMV infection and graft loss in patients presenting BKVAN, suggesting that patients with CMV reactivation should be actively screened for BKV.

## 1. Introduction

Infection caused by BK polyomavirus (BKV) is acquired in childhood. The virus infects target tissues and remains latent in the kidneys in immunocompetent hosts. Reactivation occurs in the setting of immunosuppression, specifically in cases where cellular immune responses are weakened [1]. In this scenario, replication in the graft begins, followed by expansion in the urine and eventually viremia. The interstitial tubules are the most frequent target of BKV, resulting in tubulointerstitial nephritis [2]. BKV reactivation can manifest as viruria in 30% to 40% of kidney transplant recipients (KTR), viremia in 10% to 20% or BKV-associated nephropathy in 1% to 10% of patients according to published series [3,4]. Importantly, it is estimated that BKV-associated nephropathy (BKVAN) is associated with a 50% increased risk of graft loss [5].

The main risk factor associated with the development of BKVAN is the degree of immunosuppression, with an increased risk in patients with higher levels of immunosuppression [6]. Other risk factors that have been identified are: the number of HLA mismatches, cold ischemia time, previous rejection, male sex and age [7]. Thymoglobulin induction, and treatment with tacrolimus and mycophenolate compared with cyclosporine, are factors that have also been associated with an increased risk of developing BKVAN [6,7].

Cytomegalovirus (CMV) infection is a common opportunistic infection in solid organ transplantation. CMV infection can cause direct effects following viral replication (including fever, leukopenia and thrombocytopenia with or without specific organ dysfunction) and indirect effects resulting from the action of the virus on the host’s immune response. Indirect effects include acute allograft rejection, reduced long-term graft function and an increased risk of other opportunistic infections. However, the relationship between CMV and BKV is not clear. While some studies have suggested that each virus might be a risk factor for having the other [8,9], others have found that CMV viremia may indirectly protect against subsequent BK viremia [10]. It is clear, however, that cellular immune responses play a crucial role in preventing complications of both viruses—nephropathy in the case of BVK and end-stage organ disease in CMV.

The aim of this study was to assess the relationship between BKV and CMV and their impact on BKVAN and allograft function.

## 2. Materials and Methods

### 2.1. Setting and Study Population

We conducted a retrospective study at a tertiary university referral hospital with an active kidney transplantation program (annual average: 140 kidney transplants), in Barcelona, Spain. We recorded data from all consecutive kidney transplants from 1 July 2003 to 31 December 2016 using a purpose-designed database specifically created for this study. All recipients were followed up in our hospital for at least 2 years post-transplantation, and data regarding all episodes, including episodes occurring in non-hospitalized patients, were retrospectively recorded. Collected variables included: age, gender, co-morbidities, CMV donor and recipient serostatus, prior transplants, simultaneous transplants, induction immunosuppressive regimen, maintenance immunosuppressive regimen, incidence of biopsy-proven acute allograft rejection, urological complications (nephrostomy), hemodialysis, incidence and type of opportunistic infection, incidence of biopsy-proven BKVAN and outcomes.

### 2.2. Antimicrobial Prophylaxis, Monitoring and Definitions

All recipients received prophylaxis with trimethoprim-sulfamethoxazole (160 mg/800 mg) during the first 6 months post-transplant. All patients at high risk of developing CMV infection (CMV-seronegative recipients of kidney allografts from CMV-seropositive donors) received prophylaxis with valganciclovir at a dose of 900 mg per day (adjusted for renal function) during the first 90 days post-transplant and then followed a pre-emptive strategy during the following 3 months. CMV-seropositive recipients followed a pre-emptive strategy. The pre-emptive strategy consisted of CMV viral load monitoring by real-time PCR (ELITech Group, Nanogen, Italy) every 15 days during the first 90 days post-transplantation.

BKV viral load was monitored in all recipients monthly during the first year post-transplantation by real-time PCR (ELITech Group, Nanogen, Italy). In the event that any level of BKV replication in blood was detected, subsequent viral loads were performed according to the attending physician. Recipients presenting with confirmed BKVAN were managed according to our protocol, with reduction of immunosuppressive treatment.

Presumed BKV nephropathy was defined as the persistence of high viral loads in plasma (>10^4^ copies/mL) by PCR for more than 4 weeks [3,11].

Confirmed BKVAN was defined when a renal biopsy had compatible histology (confirmed by immunohistochemistry or by in situ hybridization) according to current definitions [12].

The diagnosis of viral CMV syndrome was made when the patient had compatible clinical symptoms (fever and the presence of leukopenia or thrombocytopenia) and evidence of CMV replication in blood, determined by quantification of CMV viral load by PCR.

The first step in the case of BKVAN suspicion is reducing maintenance immunosuppression, specifically reducing tacrolimus trough levels <6 ng/mL, sirolimus < 6 ng/mL and mycophenolate mofetil/mycophenolic acid to half of the daily maintenance dose. In case of no response, we recommend that mycophenolate mofetil/mycophenolic acid is stopped and, finally, that calcineurin inhibitors are switched to sirolimus. Additionally, strategies such as intravenous immunoglobulin or leflunomide could be considered as alternative therapies.

CMV end-stage organ disease was diagnosed when a biopsy sample revealed the existence of large cells with intranuclear inclusions, isolated or associated with granular cytoplasmic inclusions that were positive for immunohistochemical staining for CMV.

Patients who suffered either from CMV and BKV simultaneously or at different time points were defined as having CMV and BKV coinfection.

We defined graft dysfunction according to the RIFLE criteria [13]: increases more than 1.5 to 2 times in serum creatinine or a decrease in glomerular filtration rate of more than 25% from baseline. Baseline serum creatinine and glomerular filtration rate were registered 30 days after the transplant procedure.

Kidney graft loss was defined as a definitive requirement for hemodialysis.

Standard kidney transplantation immunosuppressive regimen from non-high-risk donors included: Basiliximab, calcineurin inhibitors (Tacrolimus or Cyclosporine), mofetil mycophenolate/mycophenolic acid (MMF/MPS) and prednisone. From January 2013, the most used immunosuppressive therapies were Basiliximab, tacrolimus, mammalian target of Rapamycin (mTOR) inhibitors (everolimus or sirolimus) and steroids. High-immunological-risk recipients or kidney recipients from a donor after cardiac death received polyclonal antilymphocyte globulins (ATG), tacrolimus, MMF/MPS and steroids. Steroid doses: 0.5 g methylprednisolone before graft revascularization followed by 125 mg the second day, prednisone 0.5 mg/kg the third day and progressive tapering to 5 mg/day by day 90.

Tacrolimus doses: 0.1 mg/Kg before kidney transplantation and 0.15–0.2 mg/kg/day in 1 or 2 doses (combination tacrolimus–MMF/MPS) or 0.15 mg/kg/day in 1 or 2 doses (combination Tacrolimus–mTOR-i). Cyclosporine doses: 4–8 mg/kg/12 h. mTOR inhibitor doses: Everolimus (1 mg/12 h) and sirolimus (2–3 mg/day). Mycophenolate mofetil (MMF) doses: 1000 mg/12 h. Mycophenolic acid (MPS) doses: 720 mg/12 h and adjustment during evolution. Tacrolimus and cyclosporine were monitored through blood levels:

- Tacrolimus through blood levels: (a) combination tacrolimus–MMF/MPS (10–15 ng/mL 1st month, 8–12 ng/mL 2–3 months, 7–10 ng/mL after 3 months); (b) combination tacrolimus–mTOR-I (6–9 ng/mL 1st month and progressive reduction around 5 ng/mL after 3 months).
- Cyclosporine through blood levels: 1–2 weeks after kidney transplant (KT) (250–300 ng/mL), 200–250 ng/mL (3–4 week), 150–250 ng/mL (2–6 months) and 100–200 ng/mL (after 6 months)

mTOR inhibitors through blood levels: Everolimus (3–5 ng/mL) and sirolimus (3–5 ng/mL).

### 2.3. Statistical Analysis

In the comparative analysis, we used the chi-square test with Yates’ correction for categorical variables. Depending on their homogeneity, continuous variables were compared using the *t* test or Mann–Whitney test. Statistically significant variables in the univariate analysis and age and gender were entered into a multivariate model using logistic regression analysis, and the odds ratios (OR) and 95% confidence intervals (CI) were calculated. The analysis was performed using the stepwise logistic regression model of the SPSS software package (SPSS version 18.0, SPSS Inc., Chicago, IL, USA). All statistical tests were 2-tailed, and the threshold of statistical significance was set at *p* < 0.05.

## 3. Results

**Figure 1:**
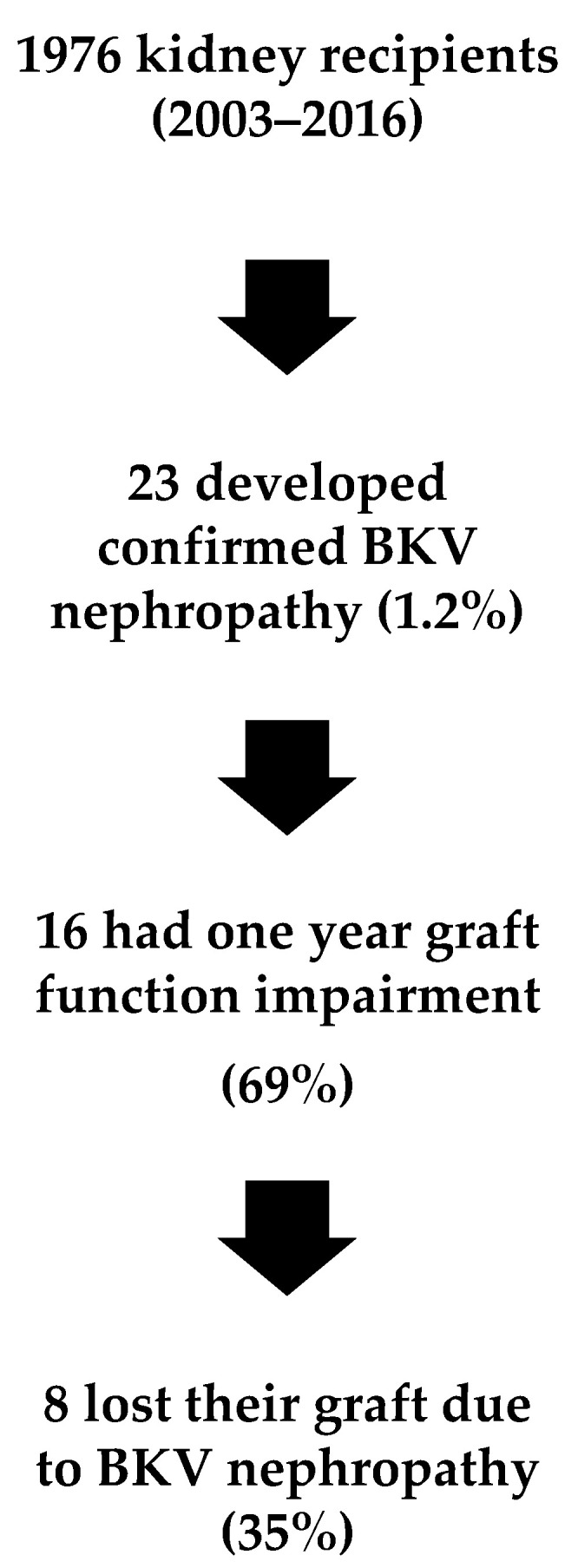
Flow of patients throughout the study. BKV: BK polyomavirus.

During the study period (14 years), 1976 patients received a kidney allograft. Mean follow-up was 1502 days (1st quartile 423—3rd quartile 1203). Two hundred and seventy-two patients lost their graft during follow-up. During the study period, 23 patients (1.2%) presented confirmed BKVAN (Figure 1). The median number of days from transplantation to BKVAN was 367 (range, 116–2011). Table 1 shows the univariate analysis of patients who presented BKVAN by presence of CMV coinfection. Patients with CMV and BKV coinfection presented higher plasma BKV viral load levels compared to patients without coinfection (*p* = 0.018). In all patients that required nephrostomy, this was due to ureteral stenosis related to early post-operative complications.

A logistic regression model of variables, analyzing predictive risk factors for BKVAN, is shown in Table 2. Diabetes mellitus (OR 3.8 95%, CI (1.4–10.5)), acute allograft rejection prior to BKVAN (6 months) (OR 2.8 95%, CI (1.1–7.6)) and nephrostomy requirement (OR 4.1 95%, CI (1.3–13)) were independently associated with the development of BKVAN. 

Regarding immunosuppressive maintenance therapies, no treatment was associated with the development of BKVAN (mycophenolic acid 1.1%, tacrolimus 1.4%, cyclosporine 1.1%, belatacept 0%, azathioprine 0%, mTOR inhibitors 1.7%), either as an individual treatment or as a combination.

Cytomegalovirus infection was diagnosed in 375 recipients (19% of all KTR); 44% of them presented as CMV disease (24 viral syndrome, 20 end-stage organ disease). None of the patients diagnosed with CMV disease presented CMV nephritis. Recipients with BKVAN presented more frequently with CMV infection compared to patients without BKVAN (39% vs. 19%, *p* = 0.02). Age > 60 years (OR 1.4 95%CI (1.1–2)), D+/R- (OR 4 95%CI (2.5–5.3)), pancreas and kidney transplantation (OR 1.8 95%CI (1.2–2.7)), acute allograft rejection (OR 2 95%CI (1.4–2.4)) and nephrostomy requirement (OR 2 95%CI (1.2–3)) were independently associated with CMV infection (Table 3).

Sixteen patients (69%) had graft dysfunction one year post-transplant. Eight patients (35%) lost their graft due to BKVAN. Multivariate analysis of risk factors associated with graft dysfunction in patients presenting with BK virus infection showed that diabetes mellitus (OR 5.2 95%CI (1.3–21.3)) was independently associated with graft dysfunction.

Table 4 shows a univariate analysis of variables related to graft loss in patients presenting with BKVAN. Cytomegalovirus infection was more frequent in patients presenting with BKVAN and graft loss (OR 2.2 95%CI (1.3–4.3)).

## 4. Discussion

In our study of a large cohort of KTR patients, we found that diabetes mellitus, acute allograft rejection and nephrostomy requirement were associated with BKVAN. Moreover, we found that CMV infection was independently associated both with graft dysfunction and graft loss in patients presenting BKVAN.

A recent systemic review showed that the most relevant risk factors for BKVAN were a regimen containing tacrolimus and acute rejection episodes [14]. Consistent with prior studies, our study also found an association between the development of BKVAN with acute allograft rejection, reflecting an augmented state of immunosuppression in these patients, and highlighting the need for active surveillance of BKV viremia. Another hypothesis could be that coinfected patients are strongly immunocompromised due to chronic mediated rejection that could not be improved despite treatment and evolves towards graft loss.

We did not find an association with any particular immunosuppressive regimen and the development of BKVAN. We also found an association with diabetes mellitus, similar to Chan BD et al. [15]—an association that most studies have not found. This fact is probably explained by the low incidence of BKVAN and the small-sized cohort studies. However, diabetes mellitus is known to be a condition that hampers immunity as well as uncontrolled disease, with high levels of glycated hemoglobin and proteinuria, which could accelerate graft loss [16]. On the other hand, the use of nephrostomy was an independent risk factor for BKVAN in our cohort. We believe that patients that require the use of nephrostomy as an early post-operative complication are patients that usually have had complications post-transplant, with an increased state of immunosuppression. The use of nephrostomy was also a risk factor for CMV infection in our cohort of patients.

On the other hand, consistent with the available data, we found that older and mismatch patients, pancreas and kidney recipients and patients with a history of prior acute allograft rejection were at higher risk of CMV infection [17,18,19]. Cytomegalovirus infection was significantly associated with the development of BKVAN and was also the only risk factor associated with graft dysfunction and graft loss in these patients. Coinfection of BKV and CMV in kidney transplant recipients has been associated with allograft dysfunction in several studies [8,20], comparable to the findings of our study. While there are some data regarding the association of CMV and BKV viremia, data correlating CMV and BKVAN are scarce. Theodoropoulos et al. found a correlation with CMV infection and BKVAN in their cohort of patients receiving Alemtuzumab as induction therapy [21]. Nevertheless, some studies have shown an inverse correlation, where BKV was associated with a lower risk of subsequent CMV infection and vice versa [10,22]. However, the design of the studies suggests that strategies to manage the reactivation of one virus, by lowering immunosuppression, might have an impact on the reactivation of the other. These associations raise the question of whether patients with CMV reactivation should be actively screened for BKV in order to prevent BKVAN. Interestingly, Reischig et al. found that patients with valganciclovir prophylaxis had an increased risk of BKV viremia compared to those receiving pre-emptive therapy [23]. The authors speculated that ganciclovir may inhibit BKV-specific T cell immunity, contributing to a higher risk of BKV. However, larger studies should confirm these findings.

Our study has several limitations; first of all, it was a study of retrospective nature. We did not evaluate several variables that have been associated with BKVAN, such as BKV viremia, ischemia injury time or the number of HLA mismatches. However, this is one of the largest cohorts exploring the association of BKV and CMV.

In conclusion, in our cohort of patients, we found that CMV infection was independently associated both with graft dysfunction and graft loss in patients presenting with BKVAN. Our study suggests that patients with CMV reactivation should be actively screened for BKV.

## Figures and Tables

**Table 1:** Univariate analysis of main characteristics of kidney recipients presenting with BK virus nephropathy by CMV coinfection.

Variable	No Coinfection *n* (%) 14 (61)	Coinfection *n* (%) 9 (39)	*p* Value
Male sex	10 (71)	5 (55)	0.656
Median age (IR)	53 (20–69)	51 (31–64)	0.543
Diabetes mellitus	5 (36)	5 (55)	1
Hepatitis C virus	2 (14)	0	0.52
HIV	0	0	
Induction therapy	13 (93)	8 (89)	1
Antilymphocyte globulin	7 (50)	5 (55)	
Basiliximab	6 (43)	3 (33)	
Immunosuppressive regimen			0.534
Cs + MMF + PDN	1	0	
FK + MMF + PDN	8	5	
mTOR + FK + PDN	5	3	
mTOR + MMF + PDN	0	1	
Prior transplantation	6 (43)	1 (11)	0.122
Double transplant (kidney and páncreas)	2 (14)	3 (33)	0.383
Median days from TX to BK nephropathy (1st, 3rd quartiles)	401 (93–870)	364 (301–508)	0.544
Postransplantation hemodialysis	1 (0.7)	1 (11)	1
Urologic reintervention	6(43)	3 (33)	1
Nephrostomy	2 (14)	2 (22)	0.654
Acute allograft rejection	5 (36)	6 (66)	0.432
Median BK viral load in blood (1st, 3rd quartiles)	709,853 (18,866–915,000)	3,636,210 (349,273–7,060,361)	0.018

CNI: calcineurin inhibitor, CMV: cytomegalovirus; HIV: human immunodeficiency virus, MMF: mycophenolate, mTOR: mammalian target of Rapamycin, PDN: prednisone, TX: transplantation, FK: Tacrolimus, Cs: Cyclosporine.

**Table 2:** Logistic regression model of variables evaluated as predictive factors of BKV nephropathy in kidney transplant recipients.

				Univariate Analysis		Multivariate Analysis	
	Category	*n*	BKV Nephropathy *n* (%)	OR (95% CI)	*p* Value	OR (95% CI)	*p* Value
Gender	Male	1144	15 (1.3)	0.8 (0.2–2.2)	0.6	0.7 (0.2–2.2)	0.645
	Female	717	8 (1.1)				
Age	Age ≥ 60	548	5 (0.9)	1.3 (0.4–3.9)	0.6	1 (0.9–1.1)	0.632
	Age < 60	1313	18 (1.4)				
Diabetes mellitus	Yes	495	10 (2)	3.6 (1.3–10)	0.01	3.8 (1.4–10.5)	0.042
	No	1297	9 (0.7)				
Hepatitis C virus infection	Yes	175	2 (1.1)	0.5 (0.06–3.8)	0.5		
	No	1679	21 (1.3)				
Induction treatment	Yes	1574	21 (1.3)	1 (0.7–1.4)	0.9		
	No	287	2 (0.7)				
Maintenance immunosuppressive therapy	Cs + MMF + PDN	67	1 (1.5)	0.9 (0.7–1.2)	0.8		
	FK + MMF + PDN	1194	13 (1)				
	Cs + mTOR + PDN	26	0				
	FK + mTOR + PDN	253	8 (3)				
	mTOR + MMF + PDN	273	1 (0.4)				
	Other	23	0				
Acute allograft rejection prior to BKVAN (6 months)	Yes	531	11 (2.1)	3 (1.1–8.2)	0.03	2.8 (1.1–7.6)	0.032
	No	1109	7 (0.6)				
Nephrostomy requirement	Yes	134	4 (3)	3.5 (1.1–12.4)	0.04	4.1 (1.3–13)	0.041
	No	1463	12 (0.8)				
CMV serology D+/R-	Yes	168	3 (1.8)	1.2 (0.3–4.1)	0.7		
	No	1376	20 (1.4)				
BK viral load in blood							

OR: odds ratios; CI: confidence intervals; D: donor; R: recipient.

**Table 3:** Logistic regression model of variables evaluated as predictive factors of CMV infection in kidney recipients.

				Univariate Analysis		Multivariate Analysis	
	Category	*n*	CMV Infection *n* (%)	OR (95% CI)	*p* Value	OR (95% CI)	*p* Value
Gender	Male	996	224 (22)	1 (0.8–1.4)	0.7	0.9 (0.7–1.1)	0.583
	Female	646	133 (20)				
Age	Age ≥ 60	468	131 (28)	1.6 (1.1–2.2)	0.003	1.4 (1.1–2)	0.021
	Age < 60	1174	226 (19)				
Diabetes mellitus	Yes	1182	238 (20)	1.2 (0.9–1.7)	0.2		
	No	443	104 (23)				
D+/R-	Yes	164	71 (43)	3.7 (2.6–5.4)	<0.001	4 (2.5–5.3)	<0.001
	No	1274	213 (17)				
HIV infection	Yes	16	1 (6)	0.5 (0.06–4)	0.5		
	No	1618	354 (22)				
Hepatitis C virus infection	Yes	165	28 (17)	1 (0.5–1.5)	0.6		
	No	1474	327 (22)				
Prior transplantation	Yes	412	80 (19)	1 (0.7–1.4)	0.5		
	No	1230	227 (22)				
Pancreas–kidney transplantation	Yes	242	63 (26)	1.8 (1.2–2.7)	0.02	1.8 (1.2–2.7)	0.003
	No	1400	294 (21)				
Induction treatment	Yes	1401	305 (22)	1 (0.9–1.1)	0.3		
	No	241	52 (22)				
Sirolimus use	Yes	451	101 (22)	0.7 (0.5–1)	0.1		
	No	1191	256 (22)				
Acute allograft rejection prior CMV infection	Yes	489	142 (29)	2 (1.4–2.5)	<0.001	2 (1.4–2.4)	<0.001
	No	1107	171 (15)				
Nephrostomy requirement	Yes	112	37 (33)	2 (1.3–3.4)	0.002	2 (1.2–3)	0.001
	No	1461	253 (17)				
Hemodyalisis post transplantation	Yes	379	94 (25)	1.4 (1.1–1.8)	0.04	1 (0.9–1.5)	0.062
	No	1198	201 (17)				

**Table 4:** Univariate analysis of variables related with graft loss in patients presenting with BKVAN.

				Univariate Analysis	
	Category	*n*	Graft Loss *n* (%)	OR (95% CI)	*p* Value
Gender	Male	15	7 (47)	0.2 (0.01–1.6)	0.1
	Female	8	1 (3)		
Age	Age ≥ 60	5	1 (20)	0.4 (0.03–4.2)	0.4
	Age < 60	18	7 (39)		
Diabetes mellitus	Yes	10	6 (60)	8 (1.1–59)	0.04
	No	13	2 (15)		
Hepatitis C virus infection	Yes	2	1 (50)	2 (0.1–37)	0.6
	No	21	7 (33)		
Induction treatment	Yes	21	8 (38)	-	
	No	2	0		
Hemodialysis requirement	Yes	2	1 (50)	2 (0.1–37)	0.6
	No	21	7 (33)		
Nephrostomy requirement	Yes	4	3 (75)	8.4 (0.7–100)	0.09
	No	19	5 (26)		
CMV infection	Yes	9	7 (78)	45 (3.4–594)	0.004
	No	14	1 (7)		
CMV disease	Yes	3	2 (67)	4.7 (0.3–62)	0.2
	No	20	6 (30)		
CMV serology D+/R-	Yes	4	1 (25)	0.6 (0.05–6.6)	0.6
	No	19	7 (37)		
Acute allograft rejection	Yes	11	5 (45)	2.5 (0.4–14.6)	0.3
	No	12	3 (25)		
Receipt of >1 pulses of 1 g intravenous methylprednisolone		7	3	0.8 (0.5–1.6)	0.9
Timoglobulin		2	1		
Receipt of rituximab		2	1		

## Data Availability

The data that support the findings of this study are available on request from the corresponding author. The data are not publicly available due to privacy or ethical restrictions.
